# Riverbed Sediments as Reservoirs of Multiple* Vibrio cholerae* Virulence-Associated Genes: A Potential Trigger for Cholera Outbreaks in Developing Countries

**DOI:** 10.1155/2017/5646480

**Published:** 2017-05-31

**Authors:** Akebe Luther King Abia, Eunice Ubomba-Jaswa, Maggy Ndombo Benteke Momba

**Affiliations:** ^1^AMBIO Environmental Management, Department of Biotechnology, Faculty of Applied and Computer Sciences, Vaal University of Technology, Vanderbijlpark 1900, South Africa; ^2^Natural Resources and the Environment, CSIR, P.O. Box 395, Pretoria 0001, South Africa; ^3^Department of Environmental, Water and Earth Science, Tshwane University of Technology, Arcadia Campus, 175 Nelson Mandela Drive, Private Bag X 680, Pretoria 0001, South Africa

## Abstract

Africa remains the most cholera stricken continent in the world as many people lacking access to safe drinking water rely mostly on polluted rivers as their main water sources. However, studies in these countries investigating the presence of* Vibrio cholerae* in aquatic environments have paid little attention to bed sediments. Also, information on the presence of virulence-associated genes (VAGs) in environmental* ctx*-negative* V. cholerae* strains in this region is lacking. Thus, we investigated the presence of* V. cholerae* VAGs in water and riverbed sediment of the Apies River, South Africa. Altogether, 120 samples (60 water and 60 sediment samples) collected from ten sites on the river (January and February 2014) were analysed using PCR. Of the 120 samples, 37 sediment and 31 water samples were positive for at least one of the genes investigated. The haemolysin gene* (hlyA)* was the most isolated gene. The cholera toxin* (ctxAB)* and non-O1 heat-stable* (stn/sto)* genes were not detected. Genes were frequently detected at sites influenced by human activities. Thus, identification of* V. cholerae* VAGs in sediments suggests the possible presence of* V. cholerae* and identifies sediments of the Apies River as a reservoir for potentially pathogenic* V. cholerae* with possible public health implications.

## 1. Introduction

Pathogenic strains of* Vibrio cholerae*, a Gram-negative bacterium, are the causative agents of the dreadful waterborne diarrhoeal disease called cholera [1]. Cholera outbreaks continue to be reported around the world, affecting over 5 million people [2] and killing over 100000 people per year [3]. Africa remains the most cholera stricken continent in the world [4], with the majority of cases and associated mortality being attributed to the lack of sanitary infrastructures and poor economic development [5]. This acute diarrhoeal disease in humans may rapidly lead to grave dehydration and even death if proper medical care, usually through oral rehydration, is not given [6, 7].

*Vibrio cholerae* has been found to survive in numerous aquatic ecosystems like rivers, lakes, streams, and oceans [8–11]. Toxigenic strains of* V. cholerae* have been found in both the water column and sediments in some of these environments [12, 13]. Monitoring of* V. cholerae* in aquatic environments usually focuses on the isolation of strains carrying the cholera toxin* (CT)* and toxin coregulated pilus* (TCP)* genes. Such strains are classified as typical O1 and O139 strains [14]. However, reports on sporadic cholera-like outbreaks have caused increased interest in non-O1 and non-O139* V. cholerae* strains [1, 12, 15–17]. Apart from the* CT* and* TCP* genes, several other genes have been reported to synergistically contribute to virulence in O1, O139, and non-O1/O139* V. cholerae*. Some of these virulence genes include the outer membrane protein* (ompW)* gene,* toxR* regulon, Zonula occludens toxin* (zot)* gene, pore-forming toxin haemolysin* (hlyA),* and the heat-stable enterotoxin* (stn/sto)* gene.

Although found to be species-specific for all* V. cholerae* [18] including nonpathogenic environmental strains, the* ompW* gene aids in increasing the adaptability of virulent strains of the organism to different environmental conditions, such as survival against bile salts in the human body [19]. The* toxR* regulon activates the transcription of the* toxT* gene, which in turn stimulates the production of proteins that activate several genes involved in virulence of* V. cholerae* [20]. This gene* (toxR)* also modulates the expression of the outer membrane proteins* OmpU* and* OmpT* [21]. During a cholera infection in mammals, the Zonula occludens toxin* (zot)* gene helps in increasing the permeability of the small intestine to* V. cholerae* [22]. It has been reported that* V. cholerae* uses the pore-forming toxin, haemolysin* (hlyA)* gene to form devices that punch holes in target eukaryotic cells, causing them to lyse during infection [23]. The* stn/sto* gene codes for the production of a heat-stable enterotoxin which disrupts fluid intake and causes the accumulation of fluid in the intestine during infection [24].

Between 1973 and 2013, South Africa reported a total of 186463 cholera cases to the World Health Organisation [25]. Over 100000 of the reported cases were in the 2001 epidemics alone, the highest number of cholera infections and deaths the country has ever experienced. Most of the cholera outbreaks in the country have been reported in rural areas without access to adequate water supplies and have been linked to polluted rivers in these areas [26]. Several studies in South Africa have reported on the presence of* V. cholerae*, including the O1 and O139 strains, in the aquatic environment [27–29]. However, these studies focused on the water column with no attention being paid to sediments. Furthermore, the investigations have focused on the detection of the* CT* gene and there is little or no information on the presence of other virulence genes, especially in environmental strains. The present study was conducted to investigate the presence of* V. cholerae* VAGs in water and riverbed sediment samples collected from the Apies River, Gauteng, South Africa. Like in many developing countries where access to safe pipe-borne water remains a challenge, the Apies River is the main source of water, for personal and household hygiene, for many communities in Gauteng. Also, many countries, including South Africa, do not include sediments quality during the monitoring of their water bodies for microbial quality. A recent study in the Apies River demonstrated that the risk of infection associated with exposure to the river could increase under conditions of sediment resuspension [30]. Identification of these virulence genes in the environment, especially in the sediments, could improve the understanding of the possible sources of the* V. cholerae* strains involved in the cholera epidemics that have affected the country for many years. Also, the detection of the VAGs in the sediments would highlight the need for many countries to include microbial sediment quality monitoring within their national water quality guidelines.

## 2. Materials and Methods

### 2.1. Study Site

**Figure 1:**
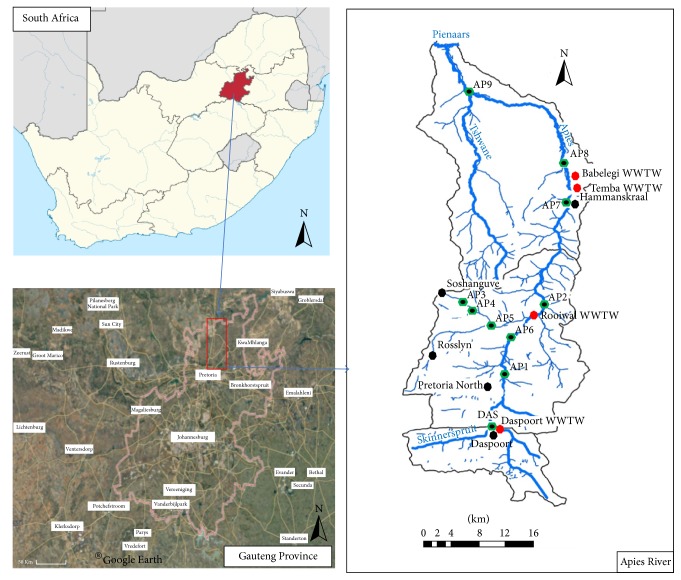
Map of the Apies River showing sampling points and wastewater treatment works (WWTW). Sites AP3, AP4, and AP5 are tributaries to the main river.

Water and sediment samples were collected from the Apies River situated in the Province of Gauteng in South Africa. A full description of the river, its tributaries, the sampling sites, and the main land uses have earlier been published [31] (Figure 1).

Briefly, the river has its source in the south of the Gauteng Province and flows northward into the Northwest Province. The portions of the river around the Pretoria CBD (Central Business District) have been canalised due to the development of the area. The portions towards the northern end of the river, as it joins the Pienaars River in the Northwest Province, have not been canalised and thus give easy access to the communities around whose inhabitants use the water for different purposes. As the river flows through rural, urban, and informal settlements around the City of Tshwane, it is used for agricultural (irrigation and animal farms), domestic, and recreational purposes [32]. A major characteristic of the land uses around the river is the presence of four main wastewater treatment works (WWTWs) (Daspoort, Rooiwal, Temba, and Babelegi) that discharge their effluents directly into the river. These WWTWs account for the greatest proportion (over 80%) of the river's total water discharge, especially during the dry winter periods [33].

### 2.2. Sample Collection

A total of 120 samples (60 water and 60 sediment samples) were collected weekly for six weeks between January and February 2014 from ten sites along the Apies River. Water samples were collected in sterile plastic bottles following standard procedures [34]. Grab sediment samples were scooped from the top 5 cm of the riverbed directly below the point at which water samples were collected and transferred into sterile plastic cups with lids. All samples were transported to the laboratory at 4°C in a cooler box with ice and analysed within 6 hours from the time of collection. All samples were collected in duplicate.

### 2.3. Sample Processing and DNA Extraction

Sediment samples were processed as previously described [35]. Fifty millilitres (50 mL) of the supernatant from resuspended sediment samples was added to an equal volume of double strength alkaline peptone water (APW) (Merck, SA). For the water samples, 50 mL of the sample from the sampling bottle was directly mixed with equal volume of the APW [36]. The enrichment step was to promote the growth of* V. cholerae* (in case where the concentration might be low) [37], thus enhancing the detection of the VAGs. The inoculated APW bottles were then incubated overnight at 37°C. After incubation, 1 mL of overnight culture was collected from the culture bottles and DNA extracted from the culture using the Instagene™ matrix (Bio-Rad, SA) following the manufacturer's instructions. Extracted DNA was stored at −20°C for use in PCR reactions the following day.

### 2.4. Identification of Virulence-Associated Genes

The identification of VAGs from the samples was done on a Corbett Life Science Rotor-Gene 6000 Cycler (Qiagen, Hilden, Germany) using three different PCR sets. Set 1 was for identification of the* ompW*,* ctxAB*,* zot,* and* tcpA* genes. Set 2 was for the* tcpI*,* hlyA,* and* toxR* genes. The last set was for the* stn/sto* genes (including the* ompW* gene as an internal control). The two multiplex PCR sets (1 and 2) were run conventionally while the last set* (stn/sto)* was run in real-time. This was because the various primers for the multiplex reactions had overlapping melting temperatures and as such could not be separated in a multiplex real-time PCR reaction. The primers used in each reaction and the size of the amplicons are given in Table 1.

A confirmed* Vibrio cholerae* strain O139 isolated from wastewater in Pretoria was used as positive control for every reaction while a reaction mixture void of template DNA was used as a No Template Control (NTC). The positive control was obtained from the microbiology laboratory of the NRE (Natural Resources and the Environment) at the Council for Scientific and Industrial Research (CSIR), Pretoria, South Africa, after being confirmed to possess the various genes of interest.

The reaction mixture for PCR Set 1 was as follows: 10 *µ*L (final concentration 1x) of 2x SensiFAST™ HRM (SF) mix; 0.5 *µ*L (F and R; 0.5 *µ*M final concentration) of each primer for* ompW*,* ctxAB*,* zot,* and* tcpA*; 1 *µ*L of nuclease free water (NF H_2_O); and 5 *µ*L of template DNA giving a total reaction volume of 25 *µ*L. For PCR Set 2, the reaction was carried out in a total volume of 25 *µ*L consisting of 2x SF mix: 10 *µ*L (final concentration 1x) and 1 *µ*L (F and R; 1 *µ*M final concentration) of each primer for* toxR* and* hlyA*; 2 *µ*L (F and R; 2 *µ*M final concentration) of each primer for* tcpI*; 2 *µ*L of NF H_2_O; and 5 *µ*L of template DNA. PCR reaction mixture for Set 3 was made up of 2x SF mix: 10 *µ*L (final concentration 1x) and 1 *µ*L (F and R; 1 *µ*M final concentration) of each primer for* stn/sto* and* ompW*; 1 *µ*L of NF H_2_O; and 5 *µ*L of template DNA for a total reaction volume of 25 *µ*L. The PCR reactions were run under optimised conditions consisting of an initial incubation step at 95°C for 50 s, followed by a 40-cycle amplification program consisting of 95°C for 10 s, 55°C for 15 s, 72°C for 25 s, and a final extension step at 72°C for 5 minutes. For PCR Set 3, the final extension was followed by preparation of a melt curve by ramping up the melting temperature from 72°C to 90°C at a ramp rate of 0.1°C at each step, holding for 90 s for premelt on the 1st step, and then holding for 2 s on each of the next steps.

### 2.5. Gel Electrophoresis

The PCR products from PCR Set 1 and Set 2 were separated by a 2.0% agarose gel electrophoresis supplemented with ethidium bromide as stain and run in a Tris-acetate-EDTA buffer. A GeneRuler™ 100 bp (Thermo Fisher Scientific, South Africa) was used as the DNA ladder. Gels were visualised under UV light and images were captured either using a digital camera or using an INGenius Syngene Bio Imaging System (Vacutec, South Africa). Once captured, the gel images were analysed using the GelAnalyzer 2010 software (http://www.gelanalyzer.com/download.html) and stored for subsequent printing.

### 2.6. Determination of Physicochemical Parameters

All physicochemical parameters were directly measured on-site during sample collection. The dissolved oxygen (DO; mg/L), electrical conductivity (EC; *μ*s/cm), pH, and temperature of the water samples were measured using an HQ40d portable Hach Multiparameter Meter (Hach, USA). Water turbidity was measured in nephelometric turbidity units (NTU) using a Eutech portable T100 turbidity meter (Eutech Instruments, Germany). Prior to being used, each instrument was calibrated using known standards from the respective suppliers.

### 2.7. Statistical Analysis

For a site to be considered positive for the gene of interest, the duplicate water or sediment samples had to be positive for the PCR assay. The occurrence of VAGs in water and sediments were compared for any statistical difference using a Kruskal Wallis test. The nonparametric Spearman's rank correlation analysis was performed to investigate any relationship between the various VAGs and the physicochemical parameters in both matrices. All tests were performed using SPSS version 20 (Statistical Package for the Social Sciences; IBM Corporation, Armonk, New York, USA) and were considered significant at *α* = 0.05.

## 3. Results

### 3.1. Distribution of* Vibrio cholerae* VAGs in Water and Sediments

A total of 60 sediment and 60 water samples were analysed for the presence of* Vibrio cholerae* VAGs using PCR following enrichment in APW. Optimised conditions for the conventional PCR reactions were verified by running the PCR product on a gel (Figure 1, in Supplementary Material available online at https://doi.org/10.1155/2017/5646480). Figure 2 is a high resolution melt (HRM) curve analysis for real-time PCR Set 3 (Supplementary Material).

Of the 120 samples analysed, 68 (37 sediment and 31 water) samples were positive for the species-specific* ompW* gene of* V. cholerae*.  Table 2 shows the distribution of the various genes investigated in this study. Overall, the VAGs were more detected in the sediments than in the water column, although this difference was not statistically significant (*p* = 0.831; *p* > 0.05). When considered individually, the percentage detection of the* ompW* gene in the sediment samples was statistically significantly higher (*p* = 0.000; *p* < 0.05) than that in the water samples. Contrary to the* ompW*, the* hlyA* gene was statistically significantly higher in the water samples (*p* = 0.012; *p* < 0.05) compared to the sediment samples. No statistically significant difference was observed between the water and the sediment samples for the detection of the* tcpA*,* toxR*,* zot,* and* tcpI* genes.

None of the samples analysed, both water and sediments, was positive for the* ctx* gene typical of O1 and O139* V. cholerae* strains. All samples analysed were also negative for the non-O1 heat-stable enterotoxin* (stn/sto)* genes. The* zot* gene was only detected in the water column at very low rates (2 out of the 120 samples analysed). The most frequently isolated VAG was the haemolysin gene (31.7% of sediment samples and 50% of water samples).

The VAGs investigated in this study were not evenly distributed amongst the sampling sites. Some genes like the* zot* gene were only detected in water samples collected at sites DAS and AP2 (Table 3). Sites AP3, AP4, and AP5 recorded the lowest prevalence of the seven virulence genes studied.

### 3.2. Distribution of Possible Virulent Genotypes in Water and Sediments

Results of the molecular profiling of the 68* V. cholerae* positive samples revealed 14 different profiles with one sample being positive for up to five of the seven VAGs investigated (Table 4).

The majority of the samples (30 of the 68 positive samples) revealed a single virulence gene. Of the 30 samples with a unique profile, the most detected genotype was the* hlyA* (23/30) genotype. The* tcpI-tcpA*,* tcpI-toxR-hlyA*,* toxR-hlyA-tcpA,* and* tcpI-toxR-hlyA-zot-tcpA* genotypes were only detected once in the entire study. Nine samples were positive for the* ompW* gene only.

### 3.3. Water Physicochemical Parameters

The water temperature during the entire sampling period ranged between 20.2°C and 28.1°C with mean temperature being 23.7°C. The DO, pH, EC, and turbidity ranged between 3.4 mg/L and 7 mg/L (mean = 5.5 mg/L), 6.6 and 8.2 (mean = 7.6), 187.8 *μ*s/cm and 654 *μ*s/cm (mean = 406.8 *μ*s/cm), and 7.1 NTU and 213 NTU (mean = 187.8 NTU), respectively.

### 3.4. Relationships between Occurrence of* V. cholerae* VAGs and Physicochemical Parameters

Within the water column, none of the genes studied showed a significant correlation with any of the physicochemical parameters measured. However, in the sediments, there was a significant positive correlation between the occurrence of the* hlyA* gene and temperature (*p* = 0.002; *p* < 0.05), the* tcpA* gene and EC (*p* = 0.013; *p* < 0.05), and the* toxR* gene and EC (*p* = 0.011; *p* < 0.05).

## 4. Discussion

### 4.1. Distribution of* Vibrio cholerae* VAGs in Water and Sediments

The main aim of the present study was to investigate the presence of* V. cholerae* VAGs in environmental samples collected from riverbed sediments of the Apies River. Several studies have shown that* V. cholerae* is present in many aquatic environments, but only a few have reported on the presence of* V. cholerae* VAGs within riverbed sediments [41–43]. In many developing countries and Sub-Saharan African countries in particular, information on the presence of* V. cholerae* strains carrying virulence gene in riverbed sediments is virtually nonexistent. These virulence genes were, however, detected in the sediments of the Apies River, South Africa. The prevalence of virulence-associated* V. cholerae* genes obtained from the environmental samples in the current study is tied with findings of previous research studies in other parts of the world [12, 42, 44]. In a study conducted on surface water in different parts of China, Li et al. [12] reported that 95.3% of the 295 non-O1/O139 environmental isolates investigated were positive for the* hlyA* gene. Ceccarelli and colleagues reported the presence of non-O1/O139* V. cholerae* isolates carrying multiple virulence genes in the sediments of the Chesapeake Bay, Maryland, USA [42]. Similarly, Bag et al. investigated 21 isolates from natural surface waters in India and found out that none of the isolates harboured the* ctx* gene but were all positive for the* hly* gene [44]. The detection of these VAGs in the absence of the* ctx* gene in various parts of the world calls for the need to further investigate the dynamics of these non-O1/O139 isolates globally, especially in countries where access to safe drinking water is still a challenge.

The detection of* V. cholerae* genes in the sediments (and water) of the Apies River could indicate the possibility of faecal pollution within this river catchment. It has previously been demonstrated that the Apies River harboured high numbers of* Escherichia coli* and* Clostridium perfringens*, good indicators of recent and long-term faecal pollution [31, 32]. The possibility of faecal pollution in the Apies River is further strengthened by the fact that* V. cholerae* VAGs were not evenly distributed between the sampling sites (Table 4). There are four wastewater treatment works (WWTWs) that discharge directly into the Apies River and their effluent is not usually of good microbial quality [32]. These WWTWs are situated upstream of sampling sites DAS (Daspoort WWTW), AP2 (Rooiwal WWTW), and AP8 (Babelegi and Temba WWTWs). The* zot* gene, for example, was only detected in the water column of sites DAS and AP2, all characterised by the presence of WWTWs. Other sites are characterised by informal settlements (AP1), agriculture (AP9), or a combination of both (AP7). Open defecation has been demonstrated to contribute to the poor microbial quality of surface water bodies [45–48]. At site AP1, there is a complete lack of sanitary facilities and the river banks and water are the only points for defecation and waste disposal. These sites (AP1, AP2, and AP8) showed the highest prevalence of the various VAGs investigated in this study. Sites that had less human influence (AP3, AP4, and AP5) showed less detection of the VAGs. Considering the fact that this study was conducted between the months of January and February, which are rainy months in South Africa, it could also be possible that the few positive samples for the* V. cholerae* VAGs identified at sites AP3, AP4, and AP5 were a result of runoff from surrounding areas (neighbouring villages without proper sanitary facilities).

The high detection of* V. cholerae* VAGs in the sediments of the Apies River also supports the fact that sediments serve as a reservoir of microorganisms where they could be resuspended, thus negatively affecting the microbial quality of the water column [49–52]. Sediments are said to provide a conducive environment for the extended survival of microorganisms, including pathogens in the aquatic ecosystem.

Although it is generally accepted that most environmental strains of* V. cholerae* are not pathogenic and thus pose no threat to human health, nonpathogenic environmental strains have been shown to evolve and have developed disease-causing potentials once within the human intestine [39]. In the present study, all the samples analysed were negative for the cholera toxin gene* (ctxAB)*, but the presence of other virulence factors indicates the possibility of infection under appropriate conditions. The high prevalence of the haemolysin gene* (hlyA)* in these environmental samples is tied with findings of Rahman et al. [53] and Ceccarelli et al. [42]. The* hlyA* gene is responsible for the production of an extracellular protein that causes extensive damage to eukaryotic cells through a hole-punching mechanism that destroys the plasma membrane of these cells [23]. The* TCP* genes responsible for mediating the production of the toxin coagulated pilus were also identified within the sediment samples in the present study. It has been shown that* TCP*, the main colonisation determinant in* V. cholerae*, is the point of attachment of the lysogenic phage CTX*φ*, the genome of which carries genetic information for the production of the cholera toxin [54]. It has been reported that* TCP* positive but non-O1/non-O139* V. cholerae* strains could be intermediates with the potential of conversion to the toxigenic forms through infection by the CTX*φ* phage [55]. This therefore means that, in the presence of this filamentous bacteriophage (CTX*φ*), nontoxigenic but* TCP* positive environmental stains of* V. cholerae* could acquire the genes for* ctx* production. Also, Faruque et al. [55] demonstrated that nontoxigenic strains of* V. cholerae* with virulence potential caused fluid accumulation within rabbit intestines suggesting that these strains were selectively enriched in the mammalian system. The detection of different possible* V. cholerae* genotypes with varying virulence genes combinations in this study (Table 4) suggests that sediments of the Apies River could be harbouring* V. cholerae* strains that may pose a potential threat to those using the waters of the Apies River for diverse purposes.

It should however be noted that the results presented in the current study are on the presence or absence of the VAGs. The enrichment step probably increased sensitivity with respect to the detection of the VAGs. Although the enrichment could have also probably ensured that the VAGs found were most likely carried in viable cells, the actual quantification of the number of cells carrying the genes could not done. This is because the APW would have favoured the multiplication of the organisms and quantitative information derived in this case would give a false impression of a high concentration of the pathogens in the samples, thus affecting the actual risk. Nevertheless, the detection of the VAGs calls for the need to further investigate the actual concentration of* V. cholerae* within the Apies River in order to determine the actual risk that the populations could be exposed to when they use untreated water from the river.

### 4.2. Correlation between Environmental Factors and the Occurrence of* V. cholerae* VAGs

Environmental factors have been found to influence the growth and survival of microorganisms in the aquatic environment. In the present study, only the* hlyA*,* tcpA,* and* toxR *genes were observed to have a significant correlation with at least one of the environmental factors. It has been shown that gene expression in* V. cholerae* is highly enhanced at temperatures of 25°C, higher pH of 8.5, high salt, and nutrient contents [56]. In the present study, however, the mean temperature and pH were 23°C and 7, respectively, which were all lower than those required for optimal expression of virulence genes in* V. cholerae* as indicated by Bhowmick and colleagues. Sediments are known to contain higher amounts of nutrients than the water column [57]. The higher nutrient concentration may favour longer survival of microorganisms in the sediments compared to the water column. This could explain the higher occurrence of the virulence genes in the sediments than in the water column and their correlation with some of the environmental factors within this matrix. The lack of correlation between most of the VAGs and the environmental parameters in this study could be due to the low variability of the physicochemical parameters as the study was conducted only during a single season. It would therefore be necessary to further study the effect of seasonal variations on the presence of these* V. cholerae* VAGs in order to better understand the relationship between the physicochemical parameters and the VAGs.

## 5. Conclusion

Based on the results obtained from the present study, we conclude that sediments of the Apies River harbour many* V. cholerae* VAGs but not the* ctx* gene. This could indicate the presence of nontoxigenic* V. cholerae* strains carrying virulence genes and hence pathogenic potentials. The presence of such strains could represent a hidden public health threat to users of the river, especially in areas where access to safe water for household uses is limited or completely absent. Also, the presence of other VAGs in the absence of the* ctx* gene of* V. cholerae* as detected in this study could stimulate further research on the possible triggers of cholera epidemics in most developing countries where toxigenic strains of the bacterium are not endemic. However, given that the present results are based on molecular detection of these VAG, it would not be possible to differentiate between* V. cholerae* and closely related species like* Vibrio mimicus*. There is therefore a need for further studies to isolate through culture and fully characterise the various* V. cholerae* strains present in sediments within South African water bodies. Also, methods such as multilocus sequence analysis (MLSA) would be necessary to obtain high resolution phenotypic differentiation between isolated* Vibrio* species.

## Supplementary Material

Gel electrophoresis (ompW, ctxAB, tcpA, zot, hlyA, toxR, tcpI genes) and High Resolution Melt (HRM) curve (ompW, stn/sto genes) of PCR products for the identification of Vibrio cholerae virulence associated genes in water and sediments of the Apies River.

## Figures and Tables

**Table 1:** Primers used for the identification of VAGs of *V. cholerae* in water and riverbed sediment samples collected from the Apies River.

Target gene	Primer sequence (5′–3′)	Amplicon size (bp)^a^	Reference
*ompW*	*F*: CACCAAGAAGGTGACTTTATTGTG	304	[18]
	*R*: GAACTTATAACCACCCGCG		
*ctxAB*	*F*: GCCGGGTTGTGGGAATGCTCCAAG	536	[38]
	*R*: GCCATACTAATTGCGGCAATCGCATG		
*Zot*	*F*: TCGCTTAACGATGGCGCGTTTT	947	[39]
	*R*: AACCCCGTTTCACTTCTACCCA		
*tcpA (El Tor)*	*F*: CACGATAAGAAAACCGGTCAAGAG	451	[39]
	*R*: CGAAAGCACCTTCTTTCACGTTG		
*tcpI*	*F*: TAGCCTTAGTTCTCAGCAGGCA	862	[39]
	*R*: GGCAATAGTGTCGAGCTCGTTA		
*hlyA*	*F*: GTGCGTATCAGCCTAGATGA	216	[40]
	R: CCAAGCTCAAAACCTGAAA		
*toxR*	*F*: CCTTCGATCCCCTAAGCAATAC	779	[39]
	*R*: AGGGTTAGCAACGATGCGTAAG		
*Stn/sto*	*F*: TCGCATTTAGCCAAACAGTAGAAA	179	[39]
	*R*: GCTGGATTGCAACATATTTCGC		

^a^bp = base pair.

**Table 2:** Distribution of *V. cholerae* virulence genes in the sediments and water column of the Apies River.

Sample type	Number of positive samples per gene (%)							
	*ompW*	*ctx*	*tcpA* ^a^	*zot*	*toxR*	*hlyA*	*tcpI*	*stn/sto*
Sediment (*n* = 60)	37 (61.7)	0 (0)	11 (18.3)	0 (0)	13 (21.7)	19 (31.7)	13 (21.7)	0 (0)
Water (*n* = 60)	31 (51.7)	0 (0)	12 (20.0)	2 (3.3)	8 (13.3)	30 (50.0)	9 (15.0)	0 (0)
*Total (n* = 120)	*68*	*0*	*23*	*2*	*21*	*49*	*22*	*0*

^a^Only the *El Tor* variant of the gene was investigated in this study.

**Table 3:** Presence/absence of the virulence genes at each of the sampling sites.

Sample type	Sample site	*ompW*	*ctx*	*tcpA*	*zot*	*toxR*	*hlyA*	*tcpI*	*stn/sto*
Sediment	DAS	+	−	+	−	+	+	+	−
	AP1	+	−	+	−	+	+	+	−
	AP2	+	−	+	−	+	+	+	−
	AP3	+	−	−	−	−	+	−	−
	AP4	+	−	+	−	−	+	−	−
	AP5	+	−	−	−	−	−	−	−
	AP6	+	−	+	−	+	+	+	−
	AP7	+	−	+	−	−	+	+	−
	AP8	+	−	+	−	+	+	+	−
	AP9	+	−	+	−	+	+	−	−

Water	DAS	+	−	+	+	−	+	+	−
	AP1	+	−	+	−	+	+	+	−
	AP2	+	−	+	+	+	+	+	−
	AP3	+	−	−	−	−	+	−	−
	AP4	+	−	+	−	−	+	+	−
	AP5	+	−	−	−	−	+	−	−
	AP6	+	−	+	−	+	+	+	−
	AP7	+	−	+	−	+	+	+	−
	AP8	+	−	+	−	+	+	+	−
	AP9	+	−	+	−	+	+	+	−

**Table 4:** Distribution of the possible *V. cholerae* virulence genotypes present in water and sediments of the Apies River.

Genotype	Number of samples
*ompW*	9
*ompW-hlyA*	23
*ompW-tcpA*	4
*ompW-toxR*	3
*ompW-tcpI-hlyA*	2
*ompW-toxR-hlyA*	6
*ompW-hlyA-tcpA*	8
*ompW-tcpI-tcpA*	1
*ompW-tcpI-toxR-hlyA*	1
*ompW-tcpI-hlyA-tcpA*	2
*ompW-toxR-hlyA-tcpA*	1
*ompW-tcpI-toxR-tcpA*	3
*ompW-tcpI-toxR-hlyA-tcpA*	4
*ompW-tcpI-toxR-hlyA-zot-tcpA*	1
