# Supplementary material for: Riverbed Sediments as Reservoirs of Multiple Vibrio cholerae Virulence-Associated Genes: A Potential Trigger for Cholera Outbreaks in Developing Countries
Source: J Environ Public Health. 2017 May 31;2017:5646480. doi: 10.1155/2017/5646480 (PMC5470021; doi:10.1155/2017/5646480)
Supplement: Supplementary file 1 — Gel electrophoresis (ompW, ctxAB, tcpA, zot, hlyA, toxR, tcpI genes) and High Resolution Melt (HRM) curve (ompW, stn/sto genes) of PCR products for the identification of Vibrio cholerae virulence associated genes in water and sediments of the Apies River. [file 5646480.f1.docx]

**Figures**

**
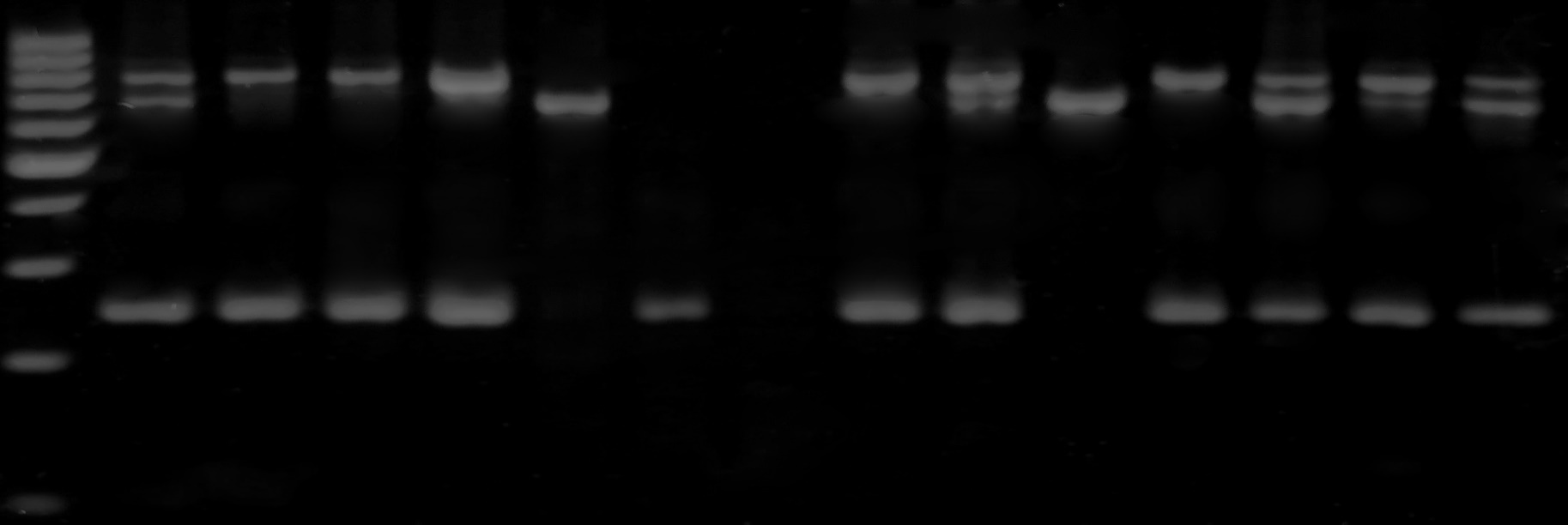
**

1000 bp

800 bp

700 bp

600 bp

500 bp

400 bp

300 bp

200 bp

100 bp

900 bp

*tcpI* (862 bp)

*toxR* (779 bp)

*hlyA* (216 bp)

Ladder +ve Control

Ladder +ve Control


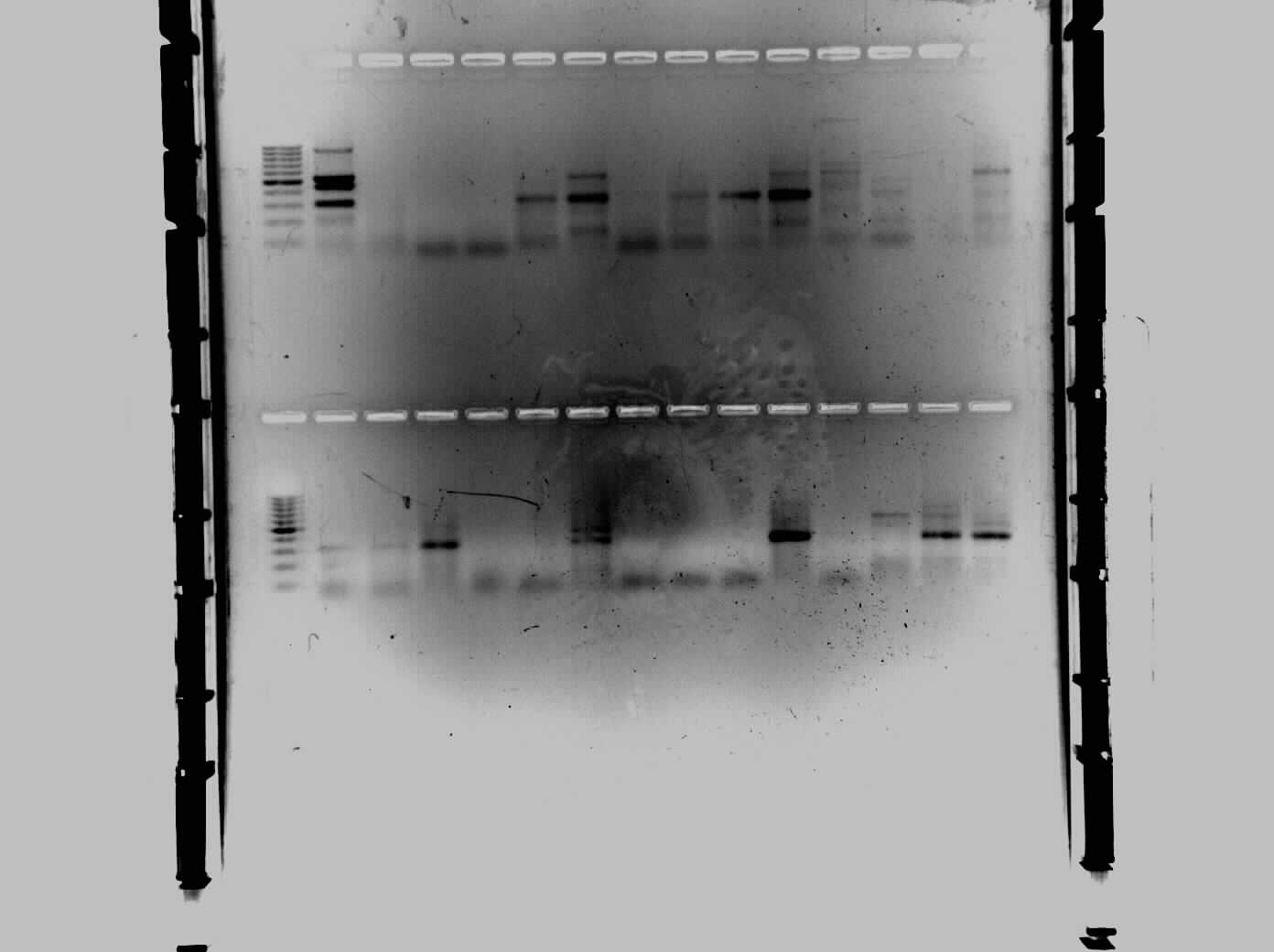


1000 bp

900 bp

800 bp

700 bp

600 bp

500 bp

400 bp

300 bp

200 bp

100 bp

*zot* (947 bp)

*ctxAB* (536 bp)

*tcpA* (415 bp)

*ompW* (304 bp)

**(a) (b)**

**Fig. 1** PCR product from (a) PCR Set 1 and (b) PCR Set 2. +ve control = *V. cholerae* 0139


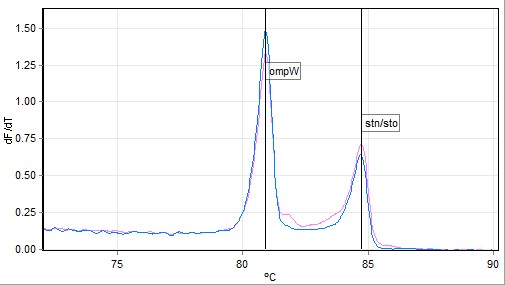


**Fig. 2** High resolution melt curve analysis for real-time PCR set 3 (*ompW* + *stn/sto*). The y-axis is the change in fluorescence with temperature while the x-axis is the temperature in degrees Celsius
